# Coexistence of Ankylosing Spondylitis and Klinefelter's Syndrome

**DOI:** 10.1155/2013/543953

**Published:** 2013-05-21

**Authors:** Şenol Kobak, Murat Yalçin, Muamer Karadeniz, Guray Oncel

**Affiliations:** ^1^Division of Rheumatology, Department of Internal Medicine, Şifa University Faculty of Medicine, 35100 Bornova/Izmir, Turkey; ^2^Division of Endocrinology, Department of Internal Medicine, Şifa University Faculty of Medicine, 35100 Bornova/Izmir, Turkey; ^3^Department of Radiology, Şifa University Faculty of Medicine, 35100 Bornova/Izmir, Turkey

## Abstract

Ankylosing spondylitis is a chronic inflammatory disease characterized by inflammatory lower back pain and morning stiffness and accompanied by spine and sacroiliac joint involvement. Klinefelter's syndrome is a genetic condition that only affects males. Affected males have an extra X chromosome. This paper reports a 30-years-old male on followup with the diagnosis of Klinefelters syndrome. The patient admitted with complaints of inflammatory lower back, and neck pain and morning stiffness and was diagnosed with ankylosing spondylitis. Nonsteroidal anti-inflammatory drug and salazopyrine treatment resulted in significant regression in his complaints.

## 1. Introduction

Klinefelters syndrome (KS) is a genetic disease affecting only males, in which there is an extra X chromosome. A male or a boy diagnosed with KS has 3 sex chromosomes (47,XXY) (classical form) or 46,XY/47XXX (mosaic form) instead of usual one X and one Y chromosomes (46,XY) [1]. Various studies have determined KS incidence to be 1 : 1000 in live male births [2]. In addition, 50% of males are not diagnosed at birth and 90% are diagnosed at after puberty period. Major findings in males with KS are microorchidism and gynecomastia, while the development of secondary sex characteristics is near normal [3]. Locomotor system findings in KS include acromicria, clinodactyly, and spinal osteoporosis. Hormone levels in KS are characterized by increased gonadotropins such as FSH and LH, increased response to gnRH, increased sex hormone binding protein (SHBP), normal and/or increased testosterone, and increased serum estrogen levels. The risk of developing autoimmune disease is higher in 47XXY males than normal 46XY males. although rare, coexistence of KS and different rheumatic diseases (systemic lupus erythematosus, rheumatoid arthritis, and systemic sclerosis) is reported in the literature [4–6]. Immune response modulation is thought to have developed upon the decrease of testosterones and increase of estrogen. We have reported coexistance of KS and ankylosing spondylitis (AS) in this paper.

## 2. Case Presentation

**Figure 1:**
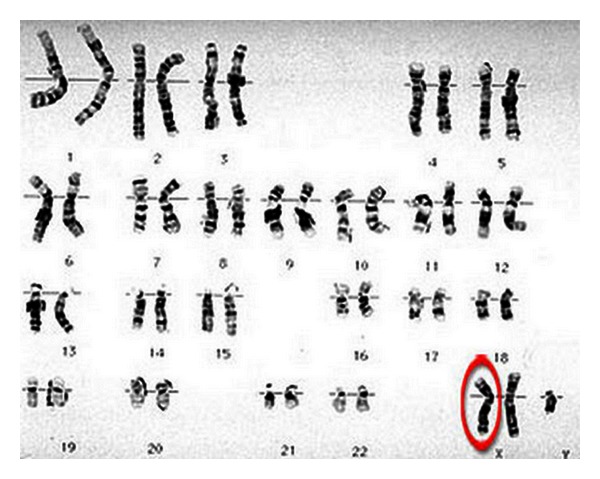
Karyogram of patient with KS suggests a 47XXY karyotype.

**Figure 2:**
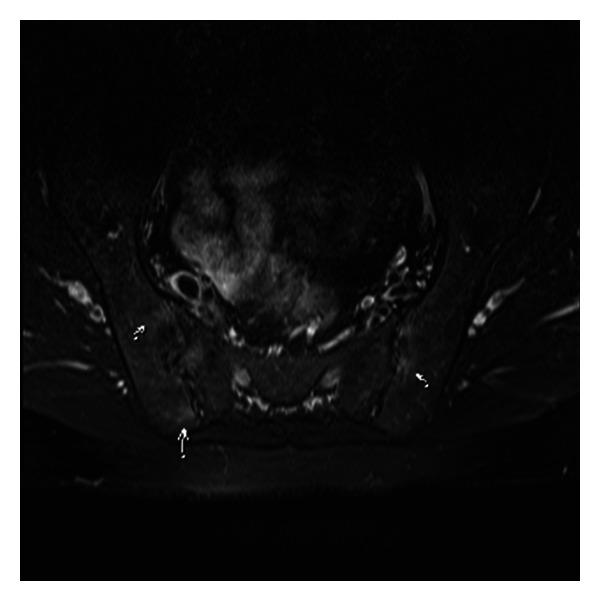
MRI of patient with KS showed active sacroiliitis.

30-years-old male patient has been on followup by the endocrinologist for about 20 years with the diagnosis of KS (Figure 1). The patient has been complaining of lower back and hip pain for about 7-8 years and was directed to the rheumatology out-patients service upon the addition of morning stiffness in recent months, lasting for more than 1 hour. He described chronic inflammatory lower back, back and neck pain, heel, pain and morning stiffness until noon and anterior uveitis exacerbation twice as well. His physical examination showed AS posture and bilateral FABERE/FADIR positivity. His scores were as follows—finger to floor distance: 12 cm, occiput to wall distance: 4 cm, chest expansion: 3 cm, Schobers test: 2 cm, BASDAI: 6.2 cm, and BASFI: 4.5 cm. Laboratory tests revealed anemia of chronic disease. Erythrocyte sedimentation rate was 37 mm/h (normal < 20 mm/h) and CRP was 1.76 mg/dL (normal < 0.5 mg/dL). HLA-B27 test was positive. Liver and renal function tests were normal. Endocrinological examination revealed decreased levels of testosterone (6.9 pg/mL, normal range: 8.6–54 pg/mL). His radiological examination showed bilateral sacroiliitis in sacroiliac joint graphy and enthesitis in lateral heel graphy (Figure 2). Edematous signal increases were observed around bilateral sacroiliac joint in magnetic resonance imaging (Figure 2). Lung graphy and abdominal USG were normal. The patient was diagnosed with AS after clinical, radiological and laboratory tests and treatment with a nonsteroidal anti-inflammatory drug and salazopyrine 2 g/day was started. Significant regression was observed in the patient's complaints and his follow-up visits are continuing.

## 3. Discussion

Endocrine system and gender dysmorphism play an important role in the development, maintenance, and regulation of immune response. In females, response to antigenic stimuli and increased prevalence of autoimmune disease are more common. These observations are perceived as the evidence of regulation of immune response by gender dysmorphism via thymic-hypothalamus-pituitary-gonadal axis [7]. The coexistance of rheumatologic and autoimmune diseases associated with gonadal dysgenesis and/or KS is reported in various cases. The coexistance of systemic lupus erythematosus, scleroderma, rheumatoid arthritis, AS, and polymyositis is reported in males with hypogonadism, and the coexistance of autoimmune thyroiditis and inflammatory bowel disease is reported in Turner syndrome. Jiménez-Balderas and colleagues detected the existence of rheumatologic/autoimmune diseases with 8 (61%) out of 13 male patients diagnosed with hypogonadism [6]. They found AS in 4 of these 8 patients, systemic lupus erythematosus in 2, juvenile rheumatoid arthritis in one and juvenile dermatomyositis in one, patient. Compared to overall health population, this was a higher rate of rheumatologic/autoimmune disease frequency, and they have suggested that the reason is the low testosterone levels and significant gonadal disorder. 

Information on the coexistance of KS and AS in the literature is limited [8, 9]. In a male case of KS and AS coexistance, Armstrong and colleagues stated that AS disease is milder clinically and radiologically [10]. Furthermore, the radiological findings of this case (mainly cervical syndesmophytes, and apophyseal joint ankylosis) were reported to be similar to radiological images of female AS cases. Tyson and colleagues also showed that AS progression in female patients was milder and it progressed primarily with cervical and osteitis pubis involvement [11]. Hart and Robinson also reported that in female AS cases, cervical involvement was more frequent [12]. Based on these data, it may be suggested that 47XXY karyotype and X chromosome observed in KS have an important role in the expression of AS disease. Besides, testosterone used in the treatment of KS may play a part in triggering and/or severe progression of AS in KS patients.

In conclusion, we have reported a rare case of AS and KS coexistance. The existence of extra X chromosome in KS may play an important role in AS expression. Besides, used in the treatment of KS, testosterone may play a part in triggering and/or severe progression of AS. Further studies are required to highlight the pathogenesis of autoimmune diseases in KS.
